# Special Issue: The Role of Cytokines in Disease

**DOI:** 10.3390/ijms27020876

**Published:** 2026-01-15

**Authors:** Svetlana Khaiboullina

**Affiliations:** Institute of Fundamental Medicine and Biology, Kazan Federal University, OpenLab, 420008 Kazan, Russia; sv.khaiboullina@gmail.com

Cytokines are key mediators of immune communication [1,2]. These small molecules play a pivotal role in the pathogenesis of a wide range of diseases caused by infection or immune dysfunction [3,4]. These secreted proteins regulate immune cell activation, differentiation, and recruitment, thereby modulating protective responses and pathological outcomes [5,6,7]. Under normal conditions, cytokines coordinate host defense and maintain tissue homeostasis [2,8,9]. However, altered cytokine production—characterized by excessive, deficient, or imbalanced secretion—can initiate or amplify inflammatory processes that directly contribute to disease development [10,11,12].

Pro-inflammatory cytokines such as IL-1β, TNF-α, and IL-6 can sustain inflammation, impair tissue integrity, and promote the progression from acute to chronic disease [13,14,15]. In contrast, anti-inflammatory cytokines (IL-10 and TGF-β) could suppress essential immune functions or facilitate immune evasion [16,17]. Abnormal cytokine production can cause immune dysregulation, leading to diverse pathological conditions including autoimmunity, fibrosis, infection, and cancer [18,19,20,21]. The central role of these cytokines in disease pathogenesis makes them excellent therapeutic targets [22,23]. Anti-TNF antibodies are currently used to treat autoimmune diseases such as rheumatoid arthritis, irritable bowel disease, psoriasis, and ankylosing spondylitis [24,25,26,27]. Furthermore, TNF-α, IL-6, and IL-17 have been suggested as therapeutic targets for psoriasis [28]. Progress in elucidating the role of cytokines in disease pathogenesis has enhanced our understanding of disease mechanisms and developing novel therapeutic strategies.

This Special Issue focusing on the role of cytokines in disease includes six publications (three original research articles and three reviews).

Samoud et al. [29] studied the interaction between cytokine signaling and antipsychotic treatment in schizophrenia. Cytokines, such as IL-6, IL-1β and INFα, can impact the neurotransmitter networks involved in schizophrenia pathophysiology. It was shown that high serum IL-6 levels are risk factors for schizophrenia [30]. This cytokine appears to decrease the survival rate of serotonergic fetal neurons in the rat brain [31]. Additionally, high IL-1β levels were found in the brains of schizophrenia patients [32]. This interleukin also contributes to the differentiation of brain cells into a dopaminergic phenotype [33]. The long-term administration of IFN-α decreased dopamine release in the rhesus monkey striatum [34]. The cytokines IL-6, IFN-γ, TNF-α, and IL-1β may contribute to schizophrenia pathogenesis by modulating tryptophan metabolism via the kynurenine pathway [35]. This study examined how psychotropic medications affect serum IFN-γ, IL-4, TGF-β1, IL-17, and BAFF levels. The authors also analyzed the relationship between cytokine levels and clinical symptoms, demonstrating that IL-17 and BAFF are linked to treatment efficacy. These findings indicate that antipsychotic drugs modulate the pro- and anti-inflammatory cytokine network. The specific involvement of IL-17 and BAFF highlights their potential as future therapeutic targets in schizophrenia. A similar conclusion was drawn by Kissi et al., who indicated that IL-17 and BAFF could be better therapeutic targets for schizophrenia compared to Th1- and Th2-type cytokines [36]. Targeting IL-17 could improve cognitive function in this group of patients [37,38].

Collectively, the findings of Samoud et al. further advance our understanding of immune dysregulation in the pathogenesis of schizophrenia. Furthermore, authors indicate that antipsychotic treatment exerts its effects—at least in part—through modulating cytokine networks. IL-17 and BAFF’s consistent association with treatment response, together with data from previous studies, supports their relevance beyond classical Th1/Th2 cytokines, highlighting them as future biomarkers and therapeutic targets. Targeting these pathways may offer novel strategies for improving clinical outcomes and cognitive function in patients with schizophrenia.

Research by Nieto-Bona et al. [39] aimed to examine the relationships between parathyroid hormone-related protein (PTHrP), the peroxisome proliferator-activated receptor gamma (PPARγ), and retinol-binding protein 4 (RBP4). PTHrP and RBP4 have been linked to poorer kidney disease outcomes [40,41]. A direct interaction between PTHrP and PPARγ, a nephroprotective nuclear receptor, has recently been reported [42].

Authors reported that PTHrP and RBP4 are elevated in pathological renal conditions. Additionally, insulin and PPARγ modulate the expression of these proteins. These findings suggest that the coordinated regulation of this system is essential for maintaining renal homeostasis; such regulation is disrupted in various pathophysiological states. PTHrP was also identified as a diagnostic marker for differentiating chronic kidney disease of unknown etiology [43]. Furthermore, it was shown that RBP4 could be a valuable predictor of chronic kidney disease [41].

Taken together, the findings of Nieto-Bona et al. underscore the importance of the PTHrP–PPARγ–RBP4 axis in regulating renal homeostasis. The data presented by the authors highlight a tightly regulated system that is disrupted in chronic kidney disease. These results suggest that targeting this pathway may offer new opportunities for diagnosing and treating renal disorders.

In the study by Dmytrzak et al. [44], the possible association between the IL-6 gene polymorphisms, rs1800797, rs1800796, and rs1800795, and the IL6R gene subunit alpha polymorphism, rs2228145, was investigated in Polish patients at risk of keloids. A keloid is a benign fibroproliferative hypertrophy of scar tissue that extends outside the original wound and invades adjacent healthy skin [45]. Keloid formation is thought to be a complex process that includes interleukin-6 signaling pathway overactivity and genetic susceptibility [46]. Data suggests that other genetic or environmental factors play a more significant role in keloid risk in this population. Future studies will identify genetic keloid risk factors.

Overall, the study suggests that the analyzed IL6 and IL6R gene polymorphisms do not play a major independent role in keloid risk in the Polish population. These findings highlight the multifactorial nature of keloid formation, indicating that additional genetic determinants contribute to disease pathogenesis. Further large-scale and multiethnic studies are warranted to identify relevant genetic risk factors and to clarify the role of cytokine signaling in keloid formation.

There are three reviews published in this Special Issue.

In the review by Korobova [47], perspectives on MDC/CCL22 regulation across diverse pathological conditions were examined, with particular emphasis on respiratory diseases and COVID-19. Macrophage-derived chemokine (MDC/CCL22) is a member of the C-C chemokine subfamily [47]. This cytokine plays a key role in T-cell maturation and migration [48]. Authors have previously demonstrated a statistically significant reduction in plasma CCL22/MDC concentrations in both acute and convalescent patients compared to controls [49]. Such authors proposed explanations for the lower CCL22/MDC level during SARS-CoV-2 infection. It was suggested that viral components could bind to MDC/CCL22, thereby impairing its activity. An alternative explanation centers on reduced dendritic cell counts and the suppression of their function.

In conclusion, the review highlights MDC/CCL22 as an important immunoregulatory chemokine, the dysregulation of which is evident in respiratory diseases, particularly COVID-19. A consistent reduction in CCL22/MDC levels, together with dendritic cell dysfunction, underscores the relevance of this chemokine in disease pathogenesis. These insights support the potential value of MDC/CCL22 as a biomarker of immune disturbance and as a target for further investigation in respiratory viral infections.

Sarb et al. [50] examined the relationship between gut–brain axis (GBA) dysregulation, serum biomarkers, and the development of cognitive disorders in inflammatory bowel disease (IBD). IBDs involve chronic gastrointestinal inflammation driven by abnormal immune responses to gut microflora [51]. The disruption of the GBA in IBDs contributes to neurobiological imbalances and affective symptoms [52]. Furthermore, the systemic inflammation associated with IBD alters the brain’s inflammatory pathways, hormonal regulation, and blood–brain barrier integrity, further influencing gut microbiota composition [53].

Emerging evidence suggests a potential link between IBD and neurodegeneration, mediated by systemic inflammation, nutritional deficiencies, GBA dysfunction, and genetic factors. The goal of this review was to identify key correlations between the gut microbiome and neurodegenerative processes, which could guide future research.

Serum markers—such as vitamins, inflammatory mediators, markers of neuronal damage, and neurotrophic factors—have been explored as predictors of neurodegenerative risk. However, current findings remain inconclusive and warrant further investigation.

In summary, the review highlights the complex interplay between gut–brain axis dysregulation, systemic inflammation, and neurobiological alterations in IBD. The emerging association between IBD and neurodegenerative processes underscores the potential contribution of immune-mediated mechanisms, microbiome imbalance, and nutritional factors to cognitive decline. Although several serum biomarkers have been proposed as predictors of neurodegenerative risk, current evidence remains inconclusive, emphasizing the need for longitudinal and mechanistic studies to identify reliable biomarkers and therapeutic targets.

In the review by Lorenz [54], a meta-analysis was conducted to assess whether polymorphisms in genes encoding interleukins and their receptors could be a risk factor for anterior cruciate ligament rupture (ACLR). Genetic factors, especially those coding for cytokines, may increase the risk of sports injuries such as ACLR [55]. A systematic search of major databases (as of 14 June 2023) identified 89 studies, of which three case–control studies (Polish, South African, and Swedish cohorts; *n* = 1282) met the meta-analysis inclusion criteria. Several polymorphisms were analyzed, including IL6 rs1800795, IL6R rs2228145, and IL1B rs16944.

Overall, IL6 rs1800795 was associated with ACLR susceptibility, while IL1B rs16944 may also contribute. In contrast, IL6R rs2228145 had no consistent association with risk of ACLR.

In conclusion, the meta-analysis supports the role for genetic variation in inflammatory cytokine genes in ACLR risk. In particular, this review highlights the contribution of the IL6 rs1800795 polymorphism and the possible role of IL1B rs16944 in ACLR pathogenesis. The absence of a consistent association with IL6R rs2228145 underscores the complexity of the genetic architecture underlying ACLR. These findings reinforce the importance of inflammatory pathways in sports injury risk and indicate the need for larger, multiethnic studies to further clarify genetic predisposition to ACL rupture.

In conclusion, this Special Issue presents a collection of research articles that advance our understanding of the role of cytokines in disease pathogenesis. These studies identify cytokines as host risk factors and indicators of treatment efficacy, underscoring their potential as both disease biomarkers and therapeutic targets.

In conclusion, this Special Issue brings together a diverse collection of research articles advancing our understanding of the multifaceted roles of cytokines in disease pathogenesis. The studies presented herein highlight cytokines as critical host-related risk factors that influence disease risk, progression, and clinical outcomes, as well as valuable indicators of therapeutic efficacy. Together, these contributions provide a foundation for future translational research aimed at refining diagnostic strategies and developing cytokine-based interventions for improved disease management.
